# Antibiotic Use and Respiratory Pathogens in Adults With Sickle Cell Disease and Acute Chest Syndrome

**DOI:** 10.1177/1060028019846118

**Published:** 2019-04-23

**Authors:** Alyssa M. Claudio, Lindsey Foltanski, Tracie Delay, Ashley Britell, Ashley Duckett, Erin R. Weeda, Nicole Bohm

**Affiliations:** 1 Medical University of South Carolina, Charleston, SC, USA; 2 South Carolina College of Pharmacy, Charleston, SC, USA; 3 Medical University of South Carolina, Charleston, SC, USA; 4 Medical University of South Carolina College of Pharmacy, Charleston, SC, USA

**Keywords:** sickle cell disease, acute chest syndrome, pneumonia, respiratory panel, atypical bacteria

## Abstract

**Background:** Acute chest syndrome (ACS) is an acute complication of sickle cell disease (SCD). Historically, the most common pathogens were *Chlamydophila pneumoniae, Mycoplasma pneumoniae*, and respiratory syncytial virus. Pediatric patients receiving guideline-adherent therapy experienced fewer ACS-related and all-cause 30-day readmissions compared with those receiving nonadherent therapy. This has not been evaluated in adults. **Objectives:** The primary objectives were to characterize antibiotic use and pathogens. The secondary objective was to assess the occurrence of readmissions associated with guideline-adherent and clinically appropriate treatment compared with regimens that did not meet those criteria. **Methods:** A retrospective cohort analysis was conducted for adults with SCD hospitalized between August 1, 2014, and July 31, 2017, with pneumonia (PNA) or ACS. The study was approved by the institutional review board. **Results:** A total of 139 patients with 255 hospitalizations were reviewed. Among 41 respiratory cultures, 3 organisms were isolated: *Cryptococcus neoformans, Pseudomonas aeruginosa*, and budding yeast. Respiratory panels were collected on 121 admissions, with 17 positive for 1 virus; all were negative for *Chlamydophila pneumoniae* and *M pneumoniae.* There were significantly more ACS-/PNA-related 7-day readmissions from patients on guideline-adherent regimens compared with nonadherent regimens (3.7% vs 0%; *P* = 0.04). **Conclusion and Relevance:** These findings challenge existing knowledge regarding the most common pathogens in adults with SCD with ACS or PNA. Routine inclusion of a macrolide may not be necessary. Future studies focused on pathogen characterization with standardized assessment are necessary to determine appropriate empirical therapy in this population.

## Introduction

Acute chest syndrome (ACS) is one of the most serious acute complications of sickle cell disease (SCD) and one of the leading causes of death and hospitalization for patients with SCD.^1^ ACS may be indistinguishable from pneumonia (PNA) because patients typically present with sudden onset of lower-respiratory tract symptoms, including cough, dyspnea, and chest pain, and a new pulmonary infiltrate on chest radiograph. The most common etiology is viral or bacterial infection.^1,2^

The National Acute Chest Syndrome Study Group performed a landmark study in 2000 to determine the causes and clinical outcomes of ACS in a population that included pediatric and adult patients.^2^ The study identified the most common infectious pathogens as *Chlamydophila pneumoniae, Mycoplasma pneumoniae*, and respiratory syncytial virus; however, a definitive cause was identified in only 38% of patients. All patients in the study were treated with a cephalosporin and erythromycin intravenously until defervescence, followed by oral antibiotics for a total of 7 to 10 days. Based on the results of this study, the National Institutes of Health Expert Panel Report 2014 recommends treatment with an intravenous cephalosporin and an oral macrolide.^1^ Most patients diagnosed with ACS are empirically treated with an antibiotic regimen that provides atypical coverage based on these guideline recommendations.

Since the aforementioned study, very few studies have examined antibiotic use in ACS, and all were conducted as observational cohorts of pediatric patients.^3^ One recent evaluation of patients with ACS aged 0 to 22 years determined that guideline-adherent therapy (ie, cephalosporin plus a macrolide) was associated with fewer hospital readmissions compared with non–guideline-adherent therapy.^4^ This has not yet been evaluated in an adult patient population.

During this period, there have also been advancements in testing for atypical bacteria. Molecular assays such as polymerase chain reactions (PCR) that identify the presence of *Chlamydophila pneumoniae* and *M pneumoniae* are incorporated in some rapid diagnostic assays for respiratory tract samples. Testing for *Legionella pneumophila* antigen, typically in the urine, can quickly identify patients with Legionnaire’s disease.^5^

The primary objectives of this study were to characterize antibiotic use and pathogens identified from hospitalized adult patients with SCD treated for PNA or ACS. The secondary objective was to assess the occurrence of readmissions associated with guideline-adherent compared with nonadherent treatment.

## Methods

This was a single-center, retrospective cohort analysis of all adult patients (age ≥ 18 years) with SCD hospitalized between August 1, 2014, and July 31, 2017, with a diagnosis of PNA or ACS at any time during hospital admission. There were no exclusion criteria for this study. This study was conducted at the Medical University of South Carolina (MUSC), a 750-bed academic medical center and was approved by the MUSC Institutional Review Board. The MUSC Clinical Database Warehouse was used to identify admissions coded as having SCD (ICD-9 282.6 and ICD-10 D57.00, D57.01, D57.02, D57.1, D57.80, D57.819) and ACS (ICD-9 517.3 and ICD-10 D57.01) or PNA (ICD-9 480-488 and ICD-10 J09-18). Any hospitalization without an ACS-/PNA-related hospitalization within the prior 30 days was considered an index admission, and all admissions in the subsequent 30 days were defined as readmissions.

Data extracted from the electronic medical record included patient demographics, length of hospital stay, antibiotic use, microbiological results, radiographic pulmonary studies, suspected concomitant infections, number of 7-day and 30-day ACS-/PNA- related and all- cause readmissions, and any adverse clinical outcomes (eg, in-hospital mortality, transfer to intensive care unit [ICU], medication-related events). Empirical antibiotic regimens were categorized as including methicillin-resistant *Staphylococcus aureus* (MRSA) activity (eg, vancomycin and linezolid), β-lactams with *Pseudomonas aeruginosa* activity (eg, piperacillin-tazobactam, cefepime, and meropenem), coverage for typical community-acquired pneumonia (CAP) pathogens (eg, cephalosporins, β-lactam/β-lactamase inhibitor combinations, and respiratory fluoroquinolones), and coverage for atypical bacteria (eg, tetracycline, fluoroquinolone, or macrolide). These same categorizations were collected at 72 hours of antibiotic therapy and at hospital discharge. Empirical regimens were deemed guideline adherent if they included a cephalosporin and a macrolide.^1^ Regimens that were not considered guideline adherent but included either a respiratory fluoroquinolone (levofloxacin or moxifloxacin) or a broad-spectrum β-lactam in addition to atypical coverage were considered to be clinically appropriate. These criteria were utilized to categorize regimens at initiation, 72 hours, and at discharge. If treatment were to have been altered based on respiratory cultures to cover possible pathogens or de-escalate to cover identified pathogens, it was considered clinically appropriate. Specific patient risk factors for antimicrobial resistance were not collected, and therefore, antibiotic regimens were not categorized according to the Infectious Disease Society of America guidelines for hospital-acquired pneumonia.^6^

Descriptive statistics were used to analyze the collected data. The χ^2^ or Fisher exact tests were used where appropriate to compare differences in the occurrence of readmission. Statistical analyses were performed using IBM SPSS v22.0 (IBM Corp, Armonk, NY, USA). An a priori *P* value of <0.05 was considered to be statistically significant.

## Results

A total of 275 admissions were identified as potential index admissions based on coding; 20 were not included because these patients were not diagnosed with ACS or PNA on chart review.

A total of 139 patients with 255 hospitalizations were included. The median patient age was 28 years (interquartile range [IQR] = 25-36); all were African American; and the majority of hospitalizations were with female patients with hemoglobin-SS disease (Table 1). The median length of hospital stay was 8 days (IQR = 5-12.5). Among the 44 respiratory cultures collected, 3 organisms were isolated: *Cryptococcus neoformans, Pseudomonas aeruginosa*, and budding yeast (1 each). Blood and urine cultures found to be positive for at least 1 organism are listed in Table 2. Of the 14 admissions with at least 1 positive blood culture, 4 were attributed to contaminated samples. Cultures from the remaining 10 patients were documented as being secondary to an infected or colonized indwelling central venous catheter. The FILMARRAY Respiratory Panel (RP), a PCR system for respiratory tract samples, was collected during 121 admissions, with 17 positive for 1 virus (Table 2). None of the RPs detected *Chlamydophila pneumoniae, M pneumoniae*, or respiratory syncytial virus. *L pneumophila* urine antigen tests were sent for 5 admissions, and all were negative.

**Table 1.** Baseline Demographics.

Age, median (IQR), years	28 (25-36)
Female, n (%)^a^	165 (65)
Sickle cell disease type, n (%)^a^	
Hemoglobin SS	228 (89.4)
Hemoglobin SC	24 (9.4)
Hemoglobin S/O Arab	2 (0.8)
Sickle beta+ Thalassemia	1 (0.4)

Abbreviation: IQR, interquartile range.

a Percentage is out of 255 hospital admissions.

**Table 2.** Microbiology Results.

Organism	Number of Isolates	Type of Infection
Respiratory (n = 44)		
*Cryptococcus neoformans*	1	PNA
*Pseudomonas aeruginosa*	1	PNA
Budding yeast	1	Contamination
Blood (n = 211)		
Coagulase-negative *Staphylococcus* sp	5	CLABSI (3), contamination (2)
Polymicrobial	3	CLABSI
MRSA	1	CLABSI
*Serratia marcescens*	1	CLABSI
*Pantoea* *agglomerans*	1	CLABSI
*Streptococcus* *pyogenes*	1	CLABSI
*Bacillus* sp, not *anthracis*	1	Contamination
*Micrococcus* sp	1	Contamination
Urine (n = 93)		
*Escherichia coli*	4	UTI
*Klebsiella* *pneumoniae*	2	UTI
*Enterococcus* *faecalis*	1	UTI
Viruses Detected by FILMARRAY Respiratory Panel (n = 121)		
Virus	Number of Positive Results	
Rhinovirus/Enterovirus	5	
Influenza virus A	4	
Parainfluenza virus type 3	3	
Human metapneumovirus	2	
Influenza B virus	2	
Coronavirus OC43	1	

Abbreviations: CLABSI, central line-associated bloodstream infection; MRSA, methicillin-resistant *Staphylococcus aureus*; PNA, pneumonia; UTI, urinary tract infection.

**Figure 1.**
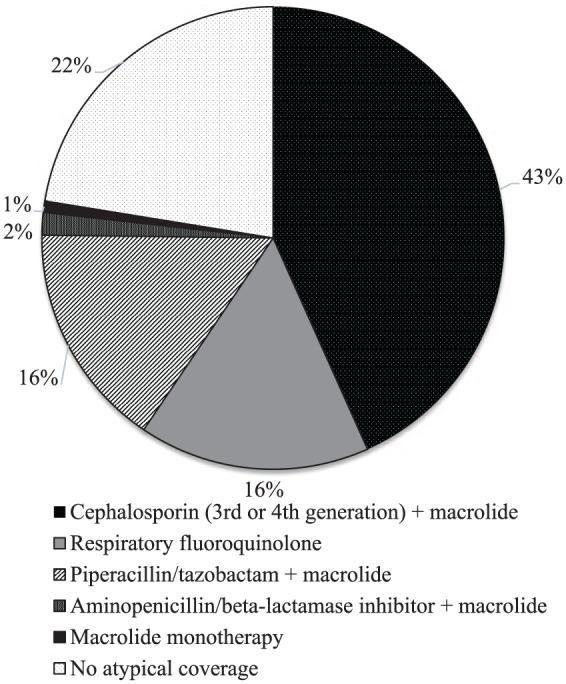
Empirical regimens.

Of 255 empirical antibiotic regimens, 197 (77%) were clinically appropriate and 110 (43%) were guideline adherent (Figure 1). Antibiotic coverage at initiation, at 72 hours, and at discharge are depicted in Table 3. Most patients were initiated on broad-spectrum antibiotics, including antipseudomonal β-lactams in 140 patients (55%) and MRSA coverage in 134 patients (53%). Although all 121 RPs collected were negative for atypical bacteria, 107 patients (88%) had atypical coverage with azithromycin or moxifloxacin continued or added to their empirical regimen for PNA/ACS. Nine patients appeared to have their atypical coverage discontinued as a result of the negative RP. The remaining 5 patients with a negative RP were never initiated on atypical coverage. The median number of inpatient days on antibiotics was 6 (IQR = 4-8), whereas the median number of total planned antibiotic days, including outpatient treatment, was 7 (IQR = 6-9). Readmissions for ACS/PNA at 7 days were significantly higher in the guideline-adherent group compared with the nonadherent group (3.7% vs 0%; *P* = 0.04); however, no other types of readmission occurrences were significantly different between groups (Table 4).

**Table 3.** Antibiotic Coverage Throughout Hospital Admission.^a^

Antibiotic Coverage	Number of Admissions, n (%), n = 255		
	Initiation	72 Hours	Discharge
MRSA	134 (53)	42 (16)	4 (2)
*Pseudomonas* sp (β-lactam)	140 (55)	67 (26)	4 (2)
CAP pathogens	120 (47)	155 (61)	81 (32)
Atypical bacteria	197 (77)	184 (72)	75 (30)

Abbreviations: CAP, community-acquired pneumonia; MRSA, methicillin-susceptible *Staphylococcus aureus*.

a Of 255 patients, 143 (56%) had completed antibiotic courses prior to the time of discharge.

**Table 4.** Readmission Results.

Type of Readmission, n (%), n = 239^a^	Guideline Adherent, n (%), n = 109	Non–Guideline Adherent, n (%), n = 130	*P* Value	Clinically Appropriate, n (%), n = 187	Insufficient Coverage, n (%), n = 52	*P* Value
ACS/Pneumonia-related 7-day	4 (3.7)	0 (0)	0.04	4 (2.1)	0 (0)	0.58
All-cause 7-day	7 (6.4)	7 (5.4)	0.73	12 (6.4)	2 (3.8)	0.74
ACS/Pneumonia-related 30-day	7 (6.4)	8 (6.2)	0.93	12 (6.4)	3 (5.8)	>0.99
All-cause 30-day	15 (13.8)	30 (23.1)	0.07	35 (18.7)	10 (19.2)	0.93

a Of the 255 acute chest syndrome (ACS) hospitalizations in this study, 239 were index encounters for ACS and 16 were readmissions treated for ACS within 30 days of these index encounters. Readmissions were compared between groups for these index encounters (ie, a total of 239 hospitalizations were in these analyses of readmissions).

Patients from 17 hospitalizations were found to have an adverse clinical outcome. Of these outcomes, 14 were escalations in level of care from the hospital floor to the ICU. One patient was transferred to the ICU for stroke and seizure activity, and 2 patients were transferred for ketamine administration. ICU transfer occurred for exchange transfusion in 4 patients and increased respiratory support requirements in 5 patients. All these patients were started on broad-spectrum antibiotics, including MRSA and *Pseudomonas* coverage, at the time of transfer to the ICU. Three of 4 of these patients were also started on azithromycin. One patient with a negative RP did not receive atypical coverage. Four patients were receiving clinically appropriate antibiotics for ACS/PNA prior to transfer to the ICU for exchange transfusion or respiratory support. One patient was transferred for respiratory failure the day after completing an 8-day course of vancomycin and cefepime. Although a sputum culture and RP were never drawn on this patient to rule out atypical pathogens, the respiratory decompensation was suspected to be secondary to opioid overdose or right heart failure. Infection was not suspected, and antibiotics were not resumed at the time of transfer. Two patients were found to have medication-related adverse events: one developed a *Clostridium difficile* infection and another experienced hives and dyspnea following ceftriaxone that resolved with diphenhydramine and ranitidine. The patient who developed *Clostridium difficile* had a positive stool PCR on the third day of vancomycin and piperacillin/tazobactam and first day of azithromycin. Cultures and RP were ordered but not collected. The patient left against medical advice the next day but was prescribed antibiotics to treat both for *Clostridium difficile* and PNA at discharge. One patient experienced in-hospital mortality attributed to septic shock secondary to an extended-spectrum β-lactamase-producing *Escherichia coli* urinary tract infection and PNA. This patient was admitted directly to the ICU and received vancomycin and piperacillin/tazobactam empirically. Based on urine culture results, the antibiotics were changed to meropenem. The patient had risk factors for multidrug-resistant organisms, including end-stage renal disease on dialysis and living in a subacute rehabilitation facility for approximately 1 month prior to admission.

## Discussion

This is the first study to characterize antibiotic use and pathogens in adult patients with SCD and ACS or PNA since the National ACS Study Group evaluated 128 adults (≥20 years) with 153 hospitalizations. This is also the first study since the use of RPs that detect *Chlamydophila* and *Mycoplasma* has become more widespread.^2^ Subsequent studies in pediatric patients have focused on total hospitalization cost, length of hospital stay, in-hospital mortality, and 7-day and 30-day readmissions.^4,7^

Currently, an intravenous cephalosporin plus an oral macrolide is recommended for ACS, regardless of patient age.^1^ Whereas only 43% of regimens in our study were guideline adherent, 77% of regimens were considered to be clinically appropriate, partially attributable to the use of a fluoroquinolone in 16% of regimens. Fluoroquinolones constitute a first-line empirical treatment option for adults with CAP with comorbidities or other risk factors for drug-resistant *Streptococcus pneumoniae*, whereas they are only recommended empirically for children in the setting of serious allergies.^8,9^ The duration of treatment observed in our study was consistent with recommendations for CAP or hospital-acquired PNA.

The overall low number of admissions with an identified respiratory pathogen in this study is not surprising because a specific cause or inciting factor is not found in many cases of ACS.^1,2^ In this study, respiratory cultures were obtained in a small percentage of patients and yielded few results. RPs identified 17 patients whose ACS or PNA could have been precipitated by a virus, with the most common being rhinovirus/enterovirus. Cessation of antibacterial agents occurred in only 2 patients. The absence of *Chlamydophila* and *Mycoplasma* is remarkable compared with the results from the National ACS Study Group, which identified 14 of 153 episodes caused by *Chlamydophila*, 8 by *Mycoplasma*, and 2 by a virus.^2^ The lower number of identified bacterial respiratory pathogens in our study could be a result of differences in diagnostic assessment and methods. The diagnostic methods were protocolized and incorporated serological testing, immunofluorescent staining, and cultures in the National ACS Study Group.^2^ However, the RP has a reported sensitivity and specificity of 95% and 99% for *Chlamydophila pneumoniae* and *M pneumoniae*, respectively, although the sensitivity for *M pneumoniae* may be slightly lower (90%) based on retrospective data.^10^ The higher frequency of viral pathogens in our study could be a result of the use of the RP, which may be more sensitive and broad than viral cultures and serological testing. Further assessment of pathogens in adults may help shape future treatment guidelines. The availability of RP results, along with consideration for additional risk factors for multidrug-resistant organisms in adults, should be considered.^3^ Of the 58 (22%) regimens that omitted atypical coverage, 40 included broad coverage for both MRSA and *P aeruginosa*, and 25 had RPs that were negative for *Chlamydophila pneumoniae* and *M pneumoniae.* Other than the patient with a fatal *E coli* infection, all patients recovered.

Overall, the results of RP testing had limited impact on antibiotic treatment at our institution other than detecting patients with influenza who were subsequently treated with oseltamivir. Although all 121 RPs collected were negative for atypical bacteria, 107 patients (88%) had atypical coverage added to their empirical regimen for PNA/ACS. Five patients with a negative RP were never initiated on atypical coverage. Only 9 patients appeared to have their atypical coverage discontinued as a result of the negative RP. If RP results are not used for this purpose, limiting the use of RPs to cases of possible or suspected influenza may offer potential cost savings.

Despite the lack of atypical bacteria identified in this study, there may be other benefits to including macrolides as part of empirical treatment for PNA/ACS. A recent study found lower in-hospital mortality in patients with bacteremic CAP who received a macrolide.^11^ Although 95% of patients in both groups had adequate coverage for the identified pathogen, the lack of benefit of fluoroquinolones suggests that the macrolide benefit may be related to immunomodulatory activity rather than spectrum of activity. The emerging role for corticosteroids in PNA also indicates that immunomodulation may be beneficial. Neither of these has been specifically evaluated in SCD patients.^12^

This is the first study to describe readmission rates after hospitalizations for ACS/PNA in adult patients. Readmission rates after pain crises in adults have been documented to be much higher than the all-cause readmission rates found in our study because 16% of patients hospitalized for acute painful episodes were readmitted within 1 week after discharge, and 50% within 1 month in a study by Ballas and Lusardi.^13^ Although both studies are single-center analyses, our readmission rate may be falsely low because patients could have been readmitted to other hospitals in the local area, making our knowledge of recent antimicrobial prescriptions or inpatient receipt incomplete. Another study in adults with vaso-occlusive crisis, including ACS, reported a 30-day readmission rate of 31%, which is closer to our 18% rate.^14^ Our all-cause readmission rates were similar to those found in pediatrics by Bundy et al^4^ for both 7-day (5.9% vs 5.2%) and 30-day (18.8% vs 13.4%) readmissions. In that study, guideline-adherent regimens were associated with significantly lower readmission rates than those involving neither a cephalosporin nor a macrolide for 30-day ACS-related and 30-day all-cause readmissions.^4^ In our study, the trend toward lower 30-day all-cause readmissions was also seen with guideline-adherent therapy compared with nonadherent therapy; however, this trend was not observed in the analysis with clinically appropriate regimens versus inappropriate regimens (Table 4). The other contrary findings in our study were that 7-day ACS-/PNA-related and 7-day all-cause readmissions were found to be numerically higher in patients on clinically appropriate treatment than those who were not on appropriate therapy. Patients on guideline-adherent regimens had significantly more 7-day ACS-/PNA-related readmissions compared with those on nonadherent regimens. We postulate that patients initiated on clinically appropriate and guideline-adherent treatment may have had more severe clinical presentations or risk factors associated with multidrug-resistant pathogens, which may have been associated with more severe underlying disease. This is just one possible explanation of the observed increase in 7-day ACS/PNA readmissions in the guideline therapy group, but we are unable to confirm this hypothesis.

One limitation of our study is that it did not aim to identify other noninfectious causes of ACS. The National ACS Study group found fat embolism, with or without infection, to be a cause in 8.8% of episodes and infarction in 16.1% of episodes.^2^ Another limitation of this study is that associations between antibiotic selection and readmission rates may have been influenced by prescribing bias. It is also unknown how patient adherence with antibiotics prescribed at discharge affected readmissions. The small number of events limits our ability to adjust for clinical factors, such as comorbid conditions, risk factors for multidrug-resistant organisms, use of simple and exchange transfusions, and mechanical ventilation, that may have affected our analysis of readmission. There is evidence suggesting that need for mechanical ventilation in adults with ACS is a predictor of mortality.^15^ Future studies are needed to identify specific risk factors for readmission for ACS because there is evidence to show that overall readmissions in adult patients with sickle cell anemia are common and may be affected by premature discharge, withdrawal syndrome, and recurrence of a new acute pain episode.^13^ The number of vaso-occlusive pain episodes requiring hospitalization in the previous year and the absence of a primary care provider have been found to be risk factors for readmission in patients with sickle cell anemia.^14^

## Conclusion and Relevance

In conclusion, the findings from this evaluation challenge existing knowledge regarding the frequency of *Chlamydophila pneumoniae, M pneumoniae*, and respiratory syncytial virus in adults with SCD with ACS or PNA. Routine inclusion of a macrolide for empirical coverage of atypical bacteria in these patients may not be necessary. Future studies focused on pathogen characterization with standardized assessment are necessary to determine appropriate empirical therapy in adult patients with SCD presenting with ACS or PNA.
